# Childhood traumatic events and adolescent overgeneral autobiographical memory: Findings in a UK cohort

**DOI:** 10.1016/j.jbtep.2014.02.004

**Published:** 2014-09

**Authors:** Catherine Crane, Jon Heron, David Gunnell, Glyn Lewis, Jonathan Evans, J. Mark G. Williams

**Affiliations:** a University of Oxford, Oxford OX3 7JX, UK; b University of Bristol, UK; c University College London, UK

**Keywords:** Memory, Adolescence, Trauma, Depression, ALSPAC

## Abstract

**Background:**

Overgeneral autobiographical memory has repeatedly been identified as a risk factor for adolescent and adult psychopathology but the factors that cause such over-generality remain unclear. This study examined the association between childhood exposure to traumatic events and early adolescent overgeneral autobiographical memory in a large population sample.

**Methods:**

Thirteen-year-olds, *n* = 5,792, participating in an ongoing longitudinal cohort study (ALSPAC) completed a written version of the Autobiographical Memory Test. Performance on this task was examined in relation to experience of traumatic events, using data recorded by caregivers close to the time of exposure.

**Results:**

Results indicated that experiencing a severe event in middle childhood increased the likelihood of an adolescent falling into the lowest quartile for autobiographical memory specificity (retrieving 0 or 1 specific memory) at age 13 by approximately 60%. The association persisted after controlling for a range of potential socio-demographic confounders.

**Limitations:**

Data on the traumatic event exposures was limited by the relatively restricted range of traumas examined, and the lack of contextual details surrounding both the traumatic event exposures themselves and the severity of children's post-traumatic stress reactions.

**Conclusions:**

This is the largest study to date of the association between childhood trauma exposure and overgeneral autobiographical memory in adolescence. Findings suggest a modest association between exposure to traumatic events and later overgeneral autobiographical memory, a psychological variable that has been linked to vulnerability to clinical depression.

## Highlights

• Overgeneral autobiographical memory has been linked to trauma in childhood.
• We examined associations between low AMT score and trauma exposure at age 13.
• A severe middle childhood life event increased likelihood of low AMT by 60%.
• This association was not appreciably attenuated after adjustment for confounders.

## 1 Introduction

Overgeneral autobiographical memory (OGM) refers to the tendency to retrieve memories of events from the personal past in a generalised way, with a lack of the event-specific details. OGM is typically assessed using the Autobiographical Memory Test (AMT, Williams & Broadbent, 1986), a task derived from Crovitz and Schiffman (1974) in which participants are given cue words and asked to retrieve a memory of a specific event: an event which occurred on one particular day, at a particular time and place, in response to each. Initially described by Williams and Broadbent (1986) in a sample of suicidal patients, a large number of subsequent studies have established that OGM is reliably observed in individuals with major depression (see Williams et al. (2007) for review and van Vreeswjik and Wilde (2004) for meta-analysis), and that OGM assessed within a depressive episode has a significant association with level of depressive symptoms at follow-up after controlling for baseline symptom severity (see Anderson, Goddard, & Powell, 2010; Gibbs & Rude, 2004; Sumner, Griffith, & Mineka, 2010; for meta- analysis). OGM has also been observed in adolescents with current major depression (Kuyken, Howell, & Dalgleish, 2006; Park, Goodyer, & Teasdale, 2002) or at high risk (Kuyken & Dalgleish, 2011) and been shown to predict depression onset in such samples (Hipwell, Sapotichne, Klostermann, Battista, & Keenan, 2011; Rawal & Rice, 2012). These findings have together led to considerable interest in the factors that may underlie the development of OGM, and its potential causal role both in determining initial onsets of depression and suicidality, and in maintaining or exacerbating existing mood disorders.

The ability to retrieve autobiographical memories develops markedly during early childhood, under the influence of emerging language abilities, self-awareness, social interaction and culture (Nelson & Fivush, 2004) and is not complete until the capacity to construct a life story is acquired in adolescence. As a result, childhood represents a sensitive period, and exposure to traumatic events during this time has been repeatedly proposed as one feature that may contribute to the development of OGM. For example in an early paper Williams (1996) suggested that OGM may represent a form of cognitive avoidance, developing initially in response to the experience of childhood traumatic events, at a time when other more active coping strategies are unavailable. According to this theory, children who have been exposed to traumatic events, particularly early in life, retain (or revert to) a retrieval style in which memory searches are terminated at the level of general event representations, reducing the negative affect that would otherwise arise in response to the recollection of specific distressing memories.

Consistent with this suggestion, a large number of subsequent studies have identified associations between various aspects of childhood trauma exposure and OGM in adults (e.g. childhood sexual abuse (CSA): Aglan, Williams, Pickles, & Hill, 2010; Hauer, Wessell, Geraerts, Merckelbach & Dalgleish, 2008; Henderson, Hargreaves, Gregory, & Williams, 2002; Kuyken & Brewin, 1995; War Trauma, Brennen et al., 2010), as well as in child and adolescent samples (e.g. childhood trauma questionnaire in adolescent inpatients, De Decker, Hermans, Raes, & Eelen, 2003; adolescents with burn injuries, Stokes, Dritschel, & Berkerian, 2004; childhood maltreatment, Valentino, Toth, & Cicchetti, 2009). However there have also been a number of failures to replicate these associations (e.g. Arntz, Meeren & Wessell, 2002; Kuyken et al., 2006; Vrielynck, Deplus, & Phillipot, 2007; Wessel, Meeren, Peters, Arntz & Merkelbach, 2001) and it remains unclear whether exposure to childhood trauma alone is sufficient to produce OGM. Further, whilst there is some evidence that earlier onset CSA is associated with more pronounced OGM (e.g. Burnside, Startup, Byatt, Rollinson, & Hill, 2004; Crane & Duggan, 2009; Hermans et al., 2004), there has been little work to systematically examine whether the risk of OGM might vary as a function of the age at which trauma exposure first occurs, particularly for traumas other than CSA, and for samples in which OGM is assessed relatively close to the time of exposure, and before the development of severe and chronic psychopathology.

Moore and Zoellner (2007) reviewed existing empirical studies examining overgeneral autobiographical memory and traumatic events, concluding that due to significant limitations and inconsistencies: “results of studies assessing potentially traumatic childhood events do not support a strong, central role for such events or related posttraumatic reactions in overgenerality…” (p. 430). The main limitations identified were that (a) childhood trauma tends to be assessed by retrospective self-report (b) many studies focus on clinical rather than community samples; (c) studies differ in whether they examine associations between OGM and exposure to potentially traumatic events themselves, or the presence of post-traumatic reactions to these events (the latter of which may be confounded with the cognitive processes that contribute to OGM); (d) there is inadequate control for current and/or past diagnoses of major depression; and finally (e) there is inadequate consideration of exposure to traumatic events which may have occurred later in life (although these are common in those exposed to childhood trauma, e.g. Coid et al., 2001). As a result Moore and Zollner argue that “well controlled studies of the association among trauma or potentially traumatic events, post-traumatic symptoms, and overgenerality are still needed…” (p. 433).

The current study utilises data from the ALSPAC birth cohort: a large and representative sample of UK children who have been studied from their mother's pregnancy until adulthood (Boyd et al., 2012; Fraser et al., 2012; Heron et al., 2012). At age 13 a written version of the AMT was included in a postal questionnaire administered to ALSPAC children (Heron et al.). Although not the main focus of the ALSPAC study, the regular questionnaires completed by caregivers included items assessing exposures to a range of potentially traumatic events, from infancy onwards. This sample enabled us to examine the associations between trauma exposure and OGM, without the need to rely on retrospective self-report. Further it allowed us to examine these associations in *young adolescents*, overcoming problems of potential adult re-traumatisation and scarring from chronic major depression, which arise when examining adult samples. Finally the comprehensive battery of measures collected for all participants in the ALSPAC cohort allowed us to control for a range of potential socio-demographic confounders as well as diagnoses of depression at ages 7.5 and 10.5 years and child depressive symptoms close to the time of AMT testing.

Although the existing literature is inconclusive, we hypothesised that a) exposure to traumatic events in childhood would be associated with increased likelihood of overgeneral autobiographical memory at age 13 years, and b) this effect would remain after excluding children who had received a diagnosis of probable clinical depression at 7.5 or 10.5 years, and after adjusting for recent low mood and socio-demographic confounders.

## 2 Method

### 2.1 Study population

The sample comprised participants from the Avon Longitudinal Study of Parents and Children (ALSPAC: Boyd et al., 2012; Fraser et al., 2012; Golding, Pembury, Jones, & ALSPAC Study Team, 2001). ALSPAC is an ongoing population-based study investigating a wide range of environmental and other influences on the health and development of children. Pregnant women resident in the former Avon Health Authority (Bristol) in South-West England, having an estimated date of delivery between 1 April 1991 and 31 December 1992, were invited to take part, resulting in a ‘core’ cohort of 14,541 pregnancies and 13,976 singletons/twins alive at 12 months of age.

A comparison of the social circumstances of enrolled mothers who completed the eight month postnatal questionnaire with all mothers in the Avon region, and all mothers with infants under one year of age nationally (using data from the 1991 census) indicated that whilst the sample was broadly representative of mothers living locally, participating ALSPAC mothers were somewhat more likely to be white, married, to live in owner-occupied accommodation and to have access to a car in the household than either all local mothers, or all mothers nationally (Fraser et al., 2012).

Three primary sources of data collection were used for this study. First, self-completion questionnaires administered at least annually to the main caregiver (usually the mother), enquiring about her own and her study child's experiences, yielded data on trauma exposure. Second, since the age of seven years the whole cohort has been invited to an annual ‘focus’ clinic for a variety of face-to-face assessments, and affective symptoms were assessed using the Mood and Feelings Questionnaire (see later) at this annual interview of the children themselves, when they were approximately 12 years 10 months old. Third, the autobiographical memory questionnaire was given as part of a generic questionnaire about teen preferences called ‘Food and Things’, sent out when the children were just over 13 years old. More detailed information on the ALSPAC study is available on the web site: http://www.alspac.bris.ac.uk.

### 2.2 Ethics statement

All aspects of the study are reviewed and approved by the ALSPAC Law and Ethics Committee, which is registered as an Institutional Review Board. Approval was also obtained from the Local Research Ethics Committees, which are governed by the Department of Health.

### 2.3 Measures

#### 2.3.1 Autobiographical Memory Test (Williams & Broadbent, 1986)

A written version of the Autobiographical Memory Test was included in a 16-page questionnaire administered to the study children when they were 13 years of age. Five positive (excited, happy, lucky, relaxed, relieved) and five negative (bored, failure, hopeless, lonely, sad) cue words were presented in pseudo-random order. Positive and negative cue words were matched for their frequency (and thus likely familiarity) in written material available for children using data from the University of Essex Children's Printed Word Database (Lovejoy, 2003; see www.essex.ac.uk/psychology/cpwd). Prior research shows good consistency between written and oral versions of the AMT, even with a significant time interval between testing sessions (*r* = 65, *n* = 90, *p* < 0.001; Raes, Hermans, Williams, & Eelen, 2006), supporting the use of the written version employed here. Further details of the AMT measure including instructions and details of the coding process are provided in Appendix 1 provided in the online supplementary materials.

Each of the ten AMT item scores was coded first as specific, extended, categoric, associate omission, before being recoded as either a specific or non-specific response and summed. The resulting variable was skewed precluding use of normal linear regression, and so it was collapsed into a binary variable for logistic regression analysis, indicating those scoring in the lowest quartile (providing at most one specific memory response) versus the remainder.

#### 2.3.2 Life events measures (adapted from Barnett, Hanna, & Parker, 1983; Brown & Harris, 1978; Coddington, 1972)

We were interested in exploring the association between OGM and severe childhood trauma of a type comparable to previous OGM research. This research has focused primarily on childhood sexual or physical abuse. Two event-exposure checklists that were administered repeatedly during childhood and completed by the child's main caregiver (usually the mother) were used to identify potentially traumatic events. One checklist focused on events affecting the study child – assessed at 18 & 30 months (toddler), 42, 57 & 69 months (early childhood), and 81 & 103 months (middle childhood) – and the other focused on events affecting the mother (also reported upon twice in toddlerhood, three times in early childhood, and twice in middle childhood, last measurement at 134 months). In each case items assessed the occurrence of each event listed during the period since the previous assessment. In order to identify relevant exposures items were classified as severe or moderate exposures, prior to any analysis of memory data. The severe exposures identified were rare and related to death of an immediate family member, physical or sexual abuse, or removal of the child from their family of origin: *being taken into care*, *being sexually abused*, *study child's parent died*, *study child's sibling died*, *mother physically cruel to her children*, *mother's partner physically cruel to her children*. The moderate exposures, whilst still rare and traumatic, were somewhat more common and somewhat more ambiguous in nature: *mother became very ill*, *family became homeless*, *mother emotionally cruel to her children*, *mother's partner emotionally cruel to her children*. Other items recorded were discounted as too ambiguous in the absence of additional contextual information (e.g. child having a shock or fright, child being separated from the mother for more than a week), not necessarily traumatic to the child (mother had a miscarriage) or likely to be so rare that they would not usefully contribute (e.g. mother convicted of an offence).

Data on the frequency of exposures (for example incidents of sexual or physical abuse), and the impact of the exposures on the study child, were not available for all items. Furthermore some items (e.g. being taken into care and being sexually abused) may be related. As a result it would have been invalid to sum exposures to create a composite ‘trauma exposure’ score. We therefore took a conservative approach and considered only the presence or absence of one or more events in each category of severity, ascribing a score to each participant (2 = severe, 1 = moderate, 0 = none) corresponding their **most severe exposure** as a toddler (up to approximately two years nine months); early childhood (from two years six months up to approximately six years, one month); and middle childhood (from five years up to approximately 11 years, two months). Age bands were approximate (and overlapping) because the reference periods for child-focused checklists and mother-focused checklists were slightly overlapping.^1^ Details of when measures where administered and the time period they referred to are shown in Appendix 2 (provided in the online supplementary materials). We did not sum trauma exposures across different developmental periods both because we anticipated that there might be recency effects and because it is highly likely that the impact of exposures changes with increasing child age.

#### 2.3.3 Development and wellbeing assessment (DAWBA, Goodman, Ford, Richards, Gatward, & Meltzer, 2000)

Mothers completed a questionnaire version of the DAWBA, which included items assessing symptoms of depression in children at 7.5 and 10.5 years of age. Questions elicited information on symptoms occurring in the *previous four weeks*, yielding two point estimates of clinical depression. Responses to these questions were reviewed by a clinical psychologist and an algorithm was applied in order to identify probable cases of depression meeting DSM-IV criteria at each time point (see Goodman et al. for further details of this measure). Probable depression was used as an exclusion criterion in subsequent analyses (see statistics section for further details). Diagnoses of PTSD were also derived at 7.5 years from DAWBA items assessing trauma exposure and post-traumatic reactions. However only one child received a probable diagnosis of PTSD and therefore we did not consider the role of PTSD further in the analysis.

#### 2.3.4 Moods and Feelings Questionnaire (Angold et al., 1995; Sharp, Goodyer, & Croudace, 2006)

The young people completed the 13-item short-form of the Moods and Feelings Questionnaire (Angold et al., 1995; Costello & Angold, 1988) during the ‘focus’ clinic held on the ALSPAC premises. The mean age of the group at this visit was 12 years 10 months (Interquartile Range: 12 yr 8 m–12 yr 11 m) i.e. typically three to four months prior to the administration of the AMT. The Moods and Feelings questionnaire comprises items measuring negative mood and was completed at a computer terminal as part of a larger battery of questions. Previous work on these data had shown that one item (*restless*) was not well-understood in this group (manuscript in preparation); hence the remaining twelve items were utilized for this analysis.

#### 2.3.5 Socio-demographic measures

Socio-demographic data was gathered from mothers through a questionnaire typically administered close to the point of entry to the study (i.e. at around the time of the child's birth). In the current analyses we adjusted for child gender, maternal age at delivery (<25/25–29/30–34/35 + years), maternal marital status at delivery (never been married/remainder), parity (first born/second born/third born or greater), housing tenure (mortgaged or owned/privately rented/subsidized/rented from council or housing association), overcrowding (more than one person per room/remainder), maternal education (no high school qualifications/high school/beyond high school), household income (quintiles of household disposable income when the child was a toddler and accounting for family size and composition, estimated housing benefits) and finally parental social class (the highest social class of either parent) at enrolment based on the Registrar General's classification of occupations, I/II (professional/managerial and technical) versus IIINM or lower (skilled non-manual/manual, semiskilled and unskilled).

### 2.4 Statistical methods

#### 2.4.1 Regression models

The raw associations between our three ordinal measures of trauma (infancy/early-childhood/middle-childhood) and a binary indicator of poor OGM (low specific memories) was estimated through a set of univariable logistic regression models. Estimates were adjusted for the potential confounding effect of the socio-demographic measures described above and then further adjusted for a proximal indicator of low mood. All analyses were repeated following the exclusion of any cases classified as having probable depression based on responses by the main caregiver in the two DAWBA questionnaires (administered at 7.5 and 10.5 years). Following these main analyses, a sensitivity analysis was carried out, testing a number of additional models using alternatively derived trauma and AMT measures. Further details on these can be found in Appendix 3 (provided in the online supplementary materials).

#### 2.4.2 Missing data treatment

Preliminary association analysis was restricted to various Complete-Case samples formed of those respondents who provided all the necessary information. Following this, Multivariate Imputation by Chained Equations (Van Buuren, Boshuizen, & Knook, 1999) was carried out using the “mi ice” routine (Royston, 2004) in Stata version 12 MP2 (Stata 10.1). The method is based on the MAR assumption that, conditional on the other data in the imputation model, there should not be systematic differences between observed and missing values for a given variable. Imputation was used to estimate missing information on trauma, socio-demographics and negative mood for those among the sample of 5792 with data on AMT. Auxiliary variables to assist with the imputation included indicators of family adversity during pregnancy (housing defects, financial problems, problems with partner relationship, poor social network and crime trouble), maternal psychopathology during pregnancy and throughout childhood, quartiles of child IQ assessed at age eight, and more proximal measures such as young person's substance use in early adolescence, maternal substance use, poor school performance, self harm and suicidal ideation. A decision was made not to attempt to impute our DAWBA exclusion criterion for the proportion of our sample that did not have this information available. This was based on the observation that the proportion of children with a probable diagnosis of depression was very low, so few cases would be missing due to missing data, and further that imputation of missing diagnoses would need to be complex given its subsequent use as an exclusion criterion. Consequently our exclusion is based only on those with an *observed* diagnosis. Logistic regression results obtained when imputing 25, 50 and 100 datasets (each with 20 cycles of regression switching) were compared, with close attention being paid to the Monte Carlo (MC) error associated with the parameter estimates, and t-statistics (White, Royston, & Wood, 2011).

## 3 Results

### 3.1 Socio-demographic characteristics

The 13-year questionnaire was sent to 10,434 of the core study participants and was returned by 6816 (65.3% response rate). Of these respondents, 5792 (85.0%) completed at least part of the AMT section (2495 males and 3297 females), ninety-five percent of whom were aged between 13 years one month and 13 years three months. Table 1 shows socio-demographic patterning across three different samples: (i) the 3542 adolescents that were not sent the questionnaire; (ii) the 4642 adolescents who were sent the questionnaire but who did not provide AMT data, either by not returning the questionnaire, *n* = 3618, or returning the questionnaire without this section completed, *n* = 1024; and (iii) the 5792 adolescents completing the AMT measure as defined above. There was strong evidence for an association, *p* < 0.001, with all socio-demographic measures chosen. As one would expect, those providing data were more likely to be from a more affluent background, and to have an older, better educated mother. Respondents were also more likely to be female.

### 3.2 Socio-demographic factors and AMT score

Table 2 shows chi-square tests of association for the socio-demographic measures and a measure of number of specific memories provided on the AMT comparing those falling into the lowest quartile to the remainder of the sample. Whilst these associations tended to be weaker than those shown in Table 1 (the associations between socio-demographics and data availability), there was strong evidence of an association between providing specific memories on the AMT and many of the socio-demographic measures chosen, typically in the same direction – those with high AMT scores tending to be female and also from a more affluent background. There was some evidence for an association between AMT score and both parity, *p* = 0.02, and maternal marital status at enrolment, *p* = 0.04.

### 3.3 AMT Performance and exposure to life events

**Fig. 1:**
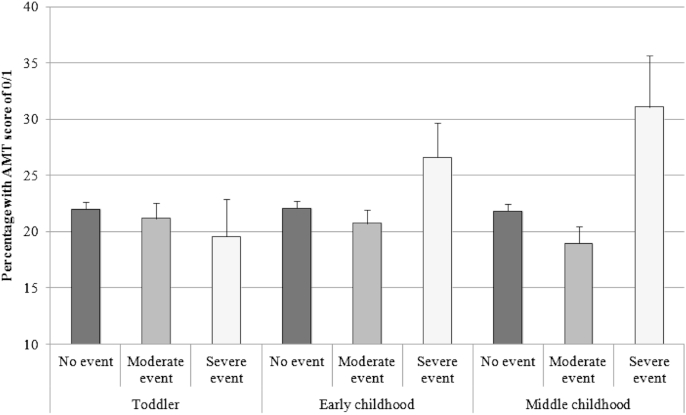
Graph showing the percentage of young people displaying overgeneral autobiographical memory (0/1 on AMT) as a function of their exposure to traumatic events in different childhood periods.

Table 3 shows results from the analyses comparing those with 0/1 specific memory (lowest quartile) to the remainder, whilst Fig. 1 shows the proportion of young people showing OGM as a function of their exposure to childhood trauma. Samples sizes for complete-case analyses vary (diminishing both with the age of the child at the time of the trauma and also as further adjustments are made for confounders) whilst the sample used for the imputed results is 5792 (without DAWBA exclusion) and 5600 (with DAWBA exclusion).

Results in the second column of Table 3 consist of simple chi-square tests. Whilst there is little evidence of a difference in rates of low specificity across levels of trauma as a toddler, there is an apparent increase in the strength association for trauma experienced in early childhood and middle childhood respectively where the rates rise steadily among those with severe trauma. Findings are very similar following exclusion of the 192 cases receiving a DSM-IV diagnosis of probable depression on the DAWBA at 7.5 or 10.5 years.

Parameter estimates from the logistic regression models (columns 2 through 4) indicate roughly a 60% increase in the odds of low memory specificity for those experiencing severe trauma in middle childhood, *OR* = 1.61, 95% *CI* = 1.06–2.47, *p* = 0.017, with this estimate changing little on adjustment for confounding variables. Estimates are lower and less stable for early-childhood trauma. Neither exclusions nor adjustment for confounders (Adjustment 1 in Table 3) or for symptoms of depression at 12 years 10 months (Adjustment 2 in Table 3) assessed by the Moods and Feelings Questionnaire (MFQ, Angold et al., 1995), made substantial changes to the findings.

Results following multiple imputation (columns 5 through 7) are in broad agreement with the complete case results discussed above. Estimates are moderately higher on exclusion of those with probable depression assessed by the DAWBA at approximately 7.5 years and 10.5 years of age, but the pattern remains.^2^

## 4 Discussion

There is ongoing interest in the potential impact of exposure to childhood traumatic events on the development of overgeneral autobiographical memory but the literature to date is inconclusive (e.g. Moore & Zoellner, 2007). The results reported here indicate that exposure to a severe event in middle childhood was associated with a modest (60%) increase in the risk of being in the lowest quartile for specific memories on the AMT at age 13 years. This effect remained largely unchanged after adjustment for a range of potential socio-demographic confounders. Exposure to severe events earlier in childhood was not associated with elevated OGM although there was some evidence of an increasing strength of association from infancy, through early childhood, to middle childhood.

The current study has a number of significant advantages, which increase confidence in the findings. First, many prior studies of the association between trauma exposure and OGM have been based on relatively small samples recruited from clinical populations or as a result of their experience of psychopathology. In contrast the current study utilised a large population based sample and made no reference to psychopathology at the point of AMT assessment. Additionally, only a few prior studies (such as Valentino et al., 2009) have been able to access data on trauma exposure other than via retrospective self-report. Although there are some inherent limitations in relying on maternal report of child trauma exposure (for example, particularly in older children, mothers may be less able to judge whether an event has been traumatic for their child) it is nevertheless an advantage to have been able to collect data throughout childhood, and relatively proximal to the exposures. As reporting occurred on 14 separate occasions during childhood, this allowed for an examination of the effects of independently reported exposure to the same traumatic events at different developmental stages.

A second strength is that we examined overgeneral autobiographical memory in young adolescents prior to the age at which most onsets of major depression occur (with analyses repeated after exclusion of adolescents who had already showed evidence of depression at either 7.5 years or 10.5 years of age, and after controlling for depressive symptoms reported 3–4 months prior to AMT testing). Thus we were able to examine the association between exposure to traumatic events and OGM in a sample largely uncontaminated by significant current or prior psychopathology, and in whom there was no potential for confounding by adult revictimisation.

Finally the availability of data on a wide range of potential confounders, including markers of more chronic stress, such as socio-economic deprivation, enabled us to examine not only social patterning of AMT responding, but also to adjust for these factors in the analyses and examine the impact of such adjustments on associations between more acute traumatic event exposure and AMT. Thus the current results provide a very conservative test of the association between trauma exposure and OGM, and in the light of this it is striking that a 60% increase in likelihood of OGM in those exposed to severe traumatic events in middle childhood was observed.

These findings counter the suggestion that exposure to traumatic events very early in life (toddler, early childhood), during the period in which the developmental shift from generic to specific retrieval is occurring, is most strongly related to later OGM (Williams, 1996). Rather, results indicated that trauma in middle childhood (approximately 5–11 years) was associated with early adolescent OGM. This has some parallels in the observation from other research that in dysphoric children OGM in response to negative cues become more apparent from age 7/8 to age 10/11, in parallel with a broader increase in memory specificity (Drummond, Dritshcel, Astall, O'Carrol, & Dalgleish, 2006). Since in the current study the AMT was not administered repeatedly throughout childhood there are at least two possible interpretations of the finding that middle childhood events had the strongest association with adolescent OGM. The first is that it is the closer proximity of the middle childhood life events to the measurement of AMT that accounts for their stronger association. Earlier life events may have had similar (or even more pronounced) effects on memory functioning, but these effects may have become less apparent by age 13, due to the passage of time and natural recovery. The second interpretation is that OGM may become increasingly prominent as a response to traumatic events as children's psychological functioning becomes more adult-like. For example, children's memory is thought to function in a similar way to adult memory by approximately seven years of age (Gathercole, 1998), corresponding to the ‘middle childhood’ period as defined in the current work. With advancing age children are also likely to be increasingly able to comprehend the meaning and implications of traumatic events, whilst at the same time having a sense of self, and their repertoire of coping strategies are still emerging and vulnerable. Future work will be required to determine whether, if exposure to equivalent traumas are considered across the life span, middle childhood remains a period in which such exposures have particular impact on later OGM, relative to similar traumas occurring earlier or later in life.

A second interesting observation was that rather than identifying a linear increase in risk of over-generality with increasing severity of event exposure in middle childhood, exposure to the particular moderately traumatic events assessed by ALSPAC was consistently associated with a slight reduction in incidence of OGM relative to those without any such exposures. One possibility is that the experience of moderate trauma may heighten the salience of emotional cue words or be associated with the construction of a more elaborate narrative structures in autobiographical memory, particularly around emotionally salient themes (resulting in facilitated recall), but be insufficient to produce the marked avoidance hypothesised to underlie OGM. A similar u-shaped relationship between exposure to adversity and other outcomes reflecting mental wellbeing has been observed in a range of contexts (e.g. Seerey, 2011), with mild trauma being suggested to increase resilience. The present findings also mirror those of studies exploring the impact of stress hormones on memory performance in animal models (e.g. Park et al., 2006). However it should be noted that more diffuse markers of socioeconomic deprivation (e.g. lower maternal social class, lower household income, overcrowding), which might be indicative of milder but more chronic stress, *were* associated with an increased likelihood of OGM. To examine the nature of the association in more detail, further investigation is required of the associations between trauma exposure and autobiographical memory specificity, including a more fine-grained analysis of exposures to both chronic stressors and acute traumas of varying severity.

Although the findings of the study are robust to control for a range of potential confounders, there are also limitations resulting from the fact that this study was an adjunct to an ongoing population based cohort study. The most prominent amongst these is that measures of trauma exposure were not optimal. For example where the child's caregiver is the main informant there are disadvantages. Caregivers may be unaware of exposures affecting their child (e.g. undisclosed sexual abuse) and/or may under-report their own maltreatment of their child. Additionally details concerning the circumstances surrounding potentially traumatic events that might have allowed for greater refinement of the exposure measure (for example the nature or duration of sexual, physical, or emotional abuse, or the circumstances of a familial death or period of homelessness) were not available. Ideally studies would use data from multiple informants to assess trauma exposure, include a wide range of carefully graded traumatic events, and include a more detailed assessment of the impact of each event on the child. However this would be an extremely challenging undertaking in a population-based study and would pose significant practical and ethical challenges.

Another issue concerns the fact that the AMT questionnaire was completed in written form by participants in their own homes, used minimal instructions and was not timed. The use of minimal instructions is likely to have resulted in the AMT indexing a particular *reporting preference* or style, rather than necessarily the *inability* to retrieve specific memories if prompted to do so. It is possible that participants may have been less willing to disclose details of events they had experienced in a written questionnaire than might have been the case using an experimenter-administered oral version, particularly if their parents were involved in questionnaire completion (i.e. the format may have accentuated an overgeneral reporting style). However it is equally plausible that the anonymity afforded by the postal questionnaire may have encouraged disclosure. Finally it has been suggested that associations between trauma exposure and OGM may be more apparent under timed conditions (Bunnell & Greenhoot, 2012). Therefore our data may underestimate the association between trauma exposure and OGM, relative to what would have been observed had the timed version of the task been employed. However the task as used in the current study may have more ecological validity, and the observation of an association even under these conditions is therefore all the more surprising.

## 5 Conclusions

Despite the above mentioned limitations the study reported here also has a number of significant strengths. There is sustained interest, but relatively little consensus, concerning the possible associations between childhood trauma and the development of OGM, and calls have been made for further research in this area. This study represents the largest to date to examine the association between childhood traumas and AMT, and the only to study such a large population sample of adolescents, entering the highest risk period for experiencing the first onset of mood disorder (Williams et al., 2012; Zisook et al., 2007). Recent research suggests that in adolescents at high risk of depression, OGM predicts subsequent onset of depression over approximately one year follow-up (Hipwell et al., 2011; Rawal & Rice, 2012). Future research is now required to determine whether level of OGM at age 13 also predicts subsequent onsets of depression and suicidality in this broader community sample (either alone or in interaction with other factors), what mechanisms might underlie these associations, and if such associations are observed how their impact might be ameliorated.

## Author note

Catherine Crane and Mark Williams are based at the Department of Psychiatry, University of Oxford. Jon Heron, David Gunnell, Jonathan Evans are based at the School of Social and Community Medicine, University of Bristol. Glyn Lewis is at the Mental Health Sciences Unit, University College London Correspondence concerning this article should be addressed to Catherine Crane, Department of Psychiatry, University of Oxford, Warneford Hospital, Warneford Lane, Oxford, OX3 7JX. +44 (0)1865 613142, email: catherine.crane@psych.ox.ac.uk

## Conflicts of interest

None of the authors have any conflict of interest with respect to the contents of this paper.

## Role of the funding source

This research was specifically funded by the Wellcome Trust grant 067797/Z/02/A awarded to Professor Mark Williams. The funder had no role in study design; in the collection, analysis and interpretation of data; in the writing of the report; and in the decision to submit the article for publication.

## Figures and Tables

**Table 1:** Demographics against data availability.

		*n*	AMT data availability			
			Not sent questionnaire	Sent but no AMT data	AMT data (1 + responses)	*X*^2^, *p*
Gender	Male	7220	1949 (55.0%)	2776 (59.8%)	2495 (43.1%)	*X*^2^ = 310.2, *p* < 0.001
	Female	6756	1593 (45.0%)	1866 (40.2%)	3297 (56.9%)	
Housing tenure	Mortgaged/owned	9559	1711 (56.6%)	3156 (72.1%)	4692 (83.4%)	*X*^2^ = 764.7, *p* < 0.001
	Private rented	1384	483 (16.0%)	444 (10.2%)	457 (8.1%)	
	Subsidized rented	2082	830 (27.5%)	775 (17.7%)	477 (8.5%)	
Parity	First born	5770	1308 (44.2%)	1761 (40.5%)	2701 (48.1%)	*X*^2^ = 97.2, *p* < 0.001
	Second born	4539	981 (33.1%)	1588 (36.5%)	1970 (35.1%)	
	Third born plus	2618	671 (22.7%)	1005 (23.1%)	942 (16.8%)	
Home overcrowding	≤1 person/room	11924	2577 (87.8%)	3991 (92.9%)	5356 (96.1%)	*X*^2^ = 207.4, *p* < 0.001
	>1 person/room	878	357 (12.2%)	305 (7.1%)	216 (3.9%)	
Maternal education	A level or higher	4392	624 (24.1%)	1282 (30.5%)	2486 (44.2%)	*X*^2^ = 604.8, *p* < 0.001
	O-level	4296	826 (32.0%)	1493 (35.5%)	1977 (35.2%)	
	< O-level	3728	1135 (43.9%)	1432 (34.0%)	1161 (20.6%)	
Household income	Top 20%	2010	178 (14.6%)	621 (17.7%)	1211 (23.3%)	*X*^2^ = 250.0, *p* < 0.001
	Middle 60%	5937	649 (53.2%)	2063 (58.8%)	3225 (61.9%)	
	Lowest 20%	1992	393 (32.2%)	827 (23.6%)	772 (14.8%)	
Social class	Professional/managerial and technical	6339	1033 (46.2%)	1938 (49.9%)	3368 (62.7%)	*X*^2^ = 239.4, *p* < 0.001
	Skilled non-manual or lower	5162	1205 (53.8%)	1949 (50.1%)	2008 (37.4%)	
Marital status at enrolment	Married (incl. divorced/widowed)	10,586	2119 (70.2%)	3579 (81.4%)	4888 (86.2%)	*X*^2^ = 327.8, *p* < 0.001
	Unmarried	2499	899 (29.8%)	819 (18.6%)	781 (13.8%)	
Maternal age at delivery	<25 years	3337	1365 (38.5%)	1095 (23.6%)	877 (15.1%)	*X*^2^ = 767.2, *p* < 0.001
	25–29	5403	1243 (35.1%)	1922 (41.4%)	2238 (38.6%)	
	30–34	3850	683 (19.3%)	1199 (25.8%)	1968 (34.0%)	
	35+	1386	251 (7.1%)	426 (9.2%)	709 (12.2%)	

**Table 2:** Demographics and AMT: Those with low memory specificity (bottom quartile) versus the rest.

		Top 3 quartiles (2–10)	Bottom quartile (0/1)	*X*^2^, *p*
Gender	Male	1817 (72.8%)	678 (27.2%)	*X*^2^ = 64.6, *p* < 0.001
	Female	2693 (81.7%)	604 (18.3%)	
Housing tenure	Mortgaged/owned	3694 (78.7%)	998 (21.3%)	*X*^2^ = 18.4, *p* < 0.001
	Private rented	352 (77.0%)	105 (23.0%)	
	Subsidized rented	335 (70.2%)	142 (29.8%)	
Parity	Firstborn	2140 (79.2%)	561 (20.8%)	*X*^2^ = 8.63, *p* = 0.013
	Second born	1538 (78.1%)	432 (21.9%)	
	Third born plus	703 (74.6%)	239 (25.4%)	
Home overcrowding	≤1 person/room	4199 (78.4%)	1157 (21.6%)	*X*^2^ = 8.74, *p* = 0.003
	>1 person/room	151 (69.9%)	65 (30.1%)	
Maternal education	A-level or higher	2052 (82.5%)	434 (17.5%)	*X*^2^ = 61.5, *p* < 0.001
	O-level	1507 (76.2%)	470 (23.8%)	
	<O-level	831 (71.6%)	330 (28.4%)	
Household income	Top 20%	997 (82.3%)	214 (17.7%)	*X*^2^ = 24.8, *p* < 0.001
	Middle 60%	2514 (78.0%)	711 (22.1%)	
	Lowest 20%	563 (72.9%)	209 (27.1%)	
Social class	Professional/managerial and technical	2725 (80.9%)	643 (19.1%)	*X*^2^ = 34.8, *p* < 0.001
	Skilled non-manual or lower	1487 (74.1%)	521 (26.0%)	
Marital status at enrolment	Married (incl. divorced/widowed)	3838 (78.5%)	1050 (21.5%)	*X*^2^ = 4.78, *p* = 0.029
	Unmarried	586 (75.0%)	195 (25.0%)	
Maternal age at delivery	<25 years	639 (72.9%)	238 (27.1%)	*X*^2^ = 18.0, *p* < 0.001
	25–29	1742 (77.8%)	496 (22.2%)	
	30–34	1556 (79.1%)	412 (20.9%)	
	35+	573 (80.8%)	136 (19.2%)	

**Table 3:** Life events versus 0/1 Specific Memory on AMT (comparison of complete case and multiple imputation results).

		Complete case analyses					Analyses based on imputed data		
		*n*	*n* (%) with 0/1 specific memories	UOR [95% CI]	Adjusted 1	Adjusted 2	UOR [95% CI]	Adjusted 1	Adjusted 2
*Without DAWBA exclusion*									
Toddler	No event	4537	998 (22.0%)	1.00 ref	1.00 ref	1.00 ref	1.00 ref	1.00 ref	1.00 ref
	Moderate event	878	186 (21.2%)	0.95 [0.80, 1.14]	0.90 [0.74, 1.09]	0.91 [0.73, 1.14]	0.96 [0.80, 1.14]	0.91 [0.76, 1.09]	0.92 [0.76, 1.10]
	Severe event	143	28 (19.6%)	0.86 [0.57, 1.31]	0.79 [0.49, 1.25]	0.78 [0.46, 1.33]	0.89 [0.58, 1.35]	0.85 [0.55, 1.29]	0.85 [0.56, 1.30]
			*p* = 0.701	*p* = 0.697	*p* = 0.349	*p* = 0.498	*p* = 0.765	*p* = 0.469	*p* = 0.503
Early childhood	No event	4115	909 (22.1%)	1.00 ref	1.00 ref	1.00 ref	1.00 ref	1.00 ref	1.00 ref
	Moderate event	1057	219 (20.7%)	0.92 [0.78, 1.09]	0.91 [0.76, 1.10]	0.96 [0.78, 1.18]	0.93 [0.79, 1.09]	0.90 [0.76, 1.06]	0.90 [0.76, 1.07]
	Severe event	203	54 (26.6%)	1.28 [0.93, 1.76]	1.24 [0.86, 1.78]	1.42 [0.96, 2.11]	1.28 [0.94, 1.76]	1.29 [0.93, 1.78]	1.31 [0.95, 1.81]
			*p* = 0.171	*p* = 0.179	*p* = 0.273	*p* = 0.189	*p* = 0.167	*p* = 0.107	*p* = 0.099
Middle childhood	No event	4458	973 (21.8%)	1.00 ref	1.00 ref	1.00 ref	1.00 ref	1.00 ref	1.00 ref
	Moderate event	697	132 (18.9%)	0.84 [0.68, 1.02]	0.80 [0.63, 1.00]	0.88 [0.68, 1.13]	0.84 [0.69, 1.03]	0.80 [0.66, 0.99]	0.81 [0.66, 1.00]
	Severe event	103	32 (31.1%)	1.61 [1.06, 2.47]	1.57 [0.96, 2.55]	1.60 [0.93, 2.73]	1.49 [0.97, 2.27]	1.38 [0.89, 2.14]	1.38 [0.89, 2.15]
			*p* = 0.014	*p* = 0.017	*p* = 0.025	*p* = 0.136	*p* = 0.037	*p* = 0.033	*p* = 0.039

*With DAWBA exclusion*									
Toddler	No event	4398	970 (22.1%)	1.00 ref	1.00 ref	1.00 ref	1.00 ref	1.00 ref	1.00 ref
	Moderate event	838	179 (21.4%)	0.96 [0.80, 1.15]	0.90 [0.73, 1.10]	0.91 [0.72, 1.15]	0.96 [0.80, 1.15]	0.92 [0.76, 1.10]	0.92 [0.76, 1.11]
	Severe event	137	28 (20.4%)	0.91 [0.60, 1.38]	0.83 [0.52, 1.33]	0.83 [0.49, 1.40]	0.92 [0.60, 1.39]	0.88 [0.57, 1.35]	0.88 [0.58, 1.35]
			*p* = 0.840	*p* = 0.697	*p* = 0.838	*p* = 0.579	*p* = 0.853	*p* = 0.564	*p* = 0.590
Early childhood	No event	4004	888 (22.2%)	1.00 ref	1.00 ref	1.00 ref	1.00 ref	1.00 ref	1.00 ref
	Moderate event	1008	210 (20.8%)	0.92 [0.78, 1.09]	0.92 [0.76, 1.11]	0.97 [0.78, 1.20]	0.92 [0.78, 1.09]	0.90 [0.75, 1.06]	0.90 [0.76, 1.07]
	Severe event	185	51 (27.6%)	1.34 [0.96, 1.86]	1.30 [0.89, 1.89]	1.47 [0.97, 2.21]	1.32 [0.95, 1.83]	1.34 [0.96, 1.88]	1.36 [0.97, 1.91]
			*p* = 0.125	*p* = 0.179	*p* = 0.135	*p* = 0.182	*p* = 0.135	*p* = 0.080	*p* = 0.075
Middle childhood	No event	4321	946 (21.9%)	1.00 ref	1.00 ref	1.00 ref	1.00 ref	1.00 ref	1.00 ref
	Moderate event	655	126 (19.2%)	0.85 [0.69, 1.05]	0.81 [0.64, 1.02]	0.88 [0.68, 1.14]	0.85 [0.70, 1.04]	0.82 [0.67, 1.01]	0.83 [0.67, 1.02]
	Severe event	94	30 (31.9%)	1.67 [1.08, 2.60]	1.68 [1.02, 2.79]	1.70 [0.97, 2.96]	1.54 [0.99, 2.39]	1.45 [0.92, 2.29]	1.46 [0.93, 2.30]
			*p* = 0.017	*p* = 0.017	*p* = 0.021	*p* = 0.106	*p* = 0.041	*p* = 0.038	*p* = 0.043

Note: AOR1 = adjusted for SES shown in all the other tables, AOR2 = further adjusted for MFQ at age 12 years 10 months. For imputation results, *n* = 5792 without DAWBA exclusion and 5600 with observed DAWBA cases excluded. For Complete Case results, sample sizes vary across trauma measures used and attenuate when adjustments for confounders are made.

## Footnotes

1 One potential concern in interpreting this data was the possibility that particular life events were clustered in particular lifetime periods, since this would confound age of exposure with type of exposure. However examining the distribution of the different life event exposures across toddlerhood, early childhood and middle childhood indicated that there was very little evidence of this sort of clustering. The only possible exception was that parental deaths were somewhat more common in middle childhood than in earlier periods, probably reflecting parental aging. However parental deaths were not the most common severe exposure at any age, making it unlikely that changes in the type of event experienced would be likely to contribute significantly to any effects of age of exposure on AMT.

2 Further sensitivity analyses were carried out to explore the impact of the decisions made regarding partial responses to both the AMT and the trauma information. The first was a complete case trauma data analysis where any partial response to the trauma questions was discarded prior to analysis and imputation. In this analysis estimates were attenuated but the conclusions were broadly the same. As anticipated, this approach resulted in a loss of information relative to the main analysis, and this was apparent in the MCerrors, which were inflated. The second sensitivity analysis was an analysis of cases in which responses were present for at least 50% of the AMT cue words, *n* = ∼4000. An apparent strengthening of the effect of severe trauma on AMT in middle childhood was observed when a complete case analysis of those with 5 + AMT was conducted. However imputation of missing data for those with <5 AMT responses produced results that were again in line with the other main analyses presented in Table 3, suggesting that this increasing of the effect was an artefact of the reduced sample size, rather than a genuine finding. Further information and tables available from the author.
