# Supplementary material for: Childhood traumatic events and adolescent overgeneral autobiographical memory: Findings in a UK cohort
Source: J Behav Ther Exp Psychiatry. 2014 Sep;45(3):330–8. doi: 10.1016/j.jbtep.2014.02.004 (PMC4053588; doi:10.1016/j.jbtep.2014.02.004)
Supplement: Supplementary file 1 [file mmc1.docx]

**Appendix 1**

AMT instructions were shortened and simplified from those typically used with adults, and no time constraint was imposed since the questionnaire was administered as part of a self-completion questionnaire (see instructions below). The use of relatively minimal instructions is supported by the findings of recent research, which suggests that minimal instructions provide a more sensitive test of memory specificity in non-clinical samples (e.g. Debeer et al., 2009).

Responses were coded as specific, extended, categoric, associate or omission, following normal conventions, by one of two data preparation assistants, who were blind to any other characteristics of the young people. Ten per cent samples were double-rated at regular intervals yielding an overall weighted kappa of .82 (excellent agreement). Further details of the coding procedure can be found in Heron et al. (2012, Appendix B). The psychometric properties of the measure in this sample have also been investigated, indicating that the test yields a unidimensional latent trait with little evidence of different dimensions based on cue valence (see Heron et al.) and little evidence that missing data followed patterns that would be expected if respondents simply completed the first few items and then got bored and moved on. Following Griffith et al., (2009), omissions were treated as genuine responses in which an individual had failed to retrieve a specific memory, rather than as data missing at random (see appendix 1 for further details of missing data treatment).

**Autobiographical Memory Instructions**

Instructions stated: “We are interested in your memory for events that have happened in your life. For each of the following words we would like you to think of an event that happened to you which the word reminds you of. The event could have happened recently (e.g. yesterday, last week) or a long time ago. It might be an important event, or a trivial event. The memory you write down should be for a **real** event. So if we said “good” – it would not be OK to say “I always enjoy a good party” because this does not mention a specific event. But it would be OK to say “I had a good time at Jane’s party” because that is a real event. Please write the real event you remember in this column. If you can’t think of an event, just leave that space blank.
