# Supplementary material for: Childhood traumatic events and adolescent overgeneral autobiographical memory: Findings in a UK cohort
Source: J Behav Ther Exp Psychiatry. 2014 Sep;45(3):330–8. doi: 10.1016/j.jbtep.2014.02.004 (PMC4053588; doi:10.1016/j.jbtep.2014.02.004)
Supplement: Supplementary file 2 [file mmc2.docx]

**Appendix 2**

| Questionnaire  Focus | Age (months) | Age  (years/months) | Time since  last measure | Reference  period  for questions | Length  of reference  period | Contributing  to measure for |
| --- | --- | --- | --- | --- | --- | --- |
| Child | 18 | 1y 6m |  | Since 6 months old | 12mn | Infancy toddler |
| Mother | 21 | 1y 9m |  | Since 8 months old | 13mn | Infancy toddler |
| Child | 30 | 2y 6m | 12mn | Since 18 months old | 12mn | Infancy toddler |
| Mother | 33 | 2y 9m | 12mn | Since 18 months old | 15mn | Infancy toddler |
| Child | 42 | 3y 6m | 12mn | In past 12 months | 12mn | Early childhood |
| Mother | 47 | 3y 11m | 14mn | Since age 2.5yrs | 17mn | Early childhood |
| Child | 57 | 4y 9m | 15mn | Since aged 3 | 21mn | Early childhood |
| Mother | 61 | 5y 1m | 14mn | Since aged 4 | 13mn | Early childhood |
| Child | 69 | 5y 9m | 12mn | In last 15 months | 15mn | Early childhood |
| Mother | 73 | 6y 1m | 12mn | Since aged 5 | 25mn | Early childhood |
| Child | 81 | 6y 9m | 12mn | Since 5^th^ birthday | 21mn | Middle childhood |
| Child | 103 | 8y 7m | 22mn | Since 7^th^ birthday | 19mn | Middle childhood |
| Mother | 110 | 9y 2m | 37mn | Since aged 6 | 37mn | Middle childhood |
| Mother | 133 | 11y 2m | 23mn | Since aged 9 | 26mn | Middle childhood |
