# Supplementary material for: Childhood traumatic events and adolescent overgeneral autobiographical memory: Findings in a UK cohort
Source: J Behav Ther Exp Psychiatry. 2014 Sep;45(3):330–8. doi: 10.1016/j.jbtep.2014.02.004 (PMC4053588; doi:10.1016/j.jbtep.2014.02.004)
Supplement: Supplementary file 3 [file mmc3.docx]

**Appendix 3**

**Sensitivity Analysis**

As there were a few, somewhat arbitrary, decisions to be made in the derivation of the exposures and outcome for these analyses, we felt it prudent to assess the degree to which any conclusions may depend on these decisions. Further details are as follows:

**(1) Trauma data.** The three trauma measures (toddler, early childhood, middle childhood) described earlier were derived from a total of *seventy* binary indicators accumulated from fourteen separate questionnaires (seven child-focused and seven caregiver-focused). Due to the large number of these measures, and the rarity of some of the events they referred to, it was not practical to run imputation models containing this fine level of detail. As consequence we imputed these data at a *coarser* level by deriving the composite trauma measures prior to imputation and inserting these measures (instead of the seventy binary indicators) into the imputation routine. To reduce the amount of observed data thrown away through this approach we opted to allow some degree of non-response when deriving our composite measures of trauma. A *valid* indicator of trauma in either toddlerhood or middle childhood needed a response to only two of the four questionnaires from that period, and similarly only three out of six questionnaire responses were needed for the early childhood period. These results were subsequently compared with the stricter approach of requiring responses to *all* the questions within each time period.

**(2) AMT data.** Our primary outcome indicates those respondents who fell into the lowest quartile on the AMT, giving no more than one specific response. Previous work (Heron et al., 2012) has included all respondents to the AMT who provided an answer to at least one of the ten cue words. Rather than assuming a non-response to be missing data containing no useful information, gaps were assumed to be the poorest response possible (below *associate*) as has been done in previous studies (Griffith et al., 2009). Here we make the implicit assumption that in line with the instructions to participants, an omission was indicative of the young person being unable to give a proper response. As we have observed previously that rates of omission were higher for cue words likely to be more rarely experienced in this age group (e.g. *hopeless*) we feel this assumption can be justified. Furthermore we feel this is more appropriate than a missing at random (MAR) assumption which would state that responses that *are* obtained were a good representation of the full set of ten that could have been collected. Some previous studies have discounted cases where participants have failed to respond to more than 50% of cue words. We conducted a further sensitivity analysis restricted to those who gave five or more responses, with imputation used to boost the sample back to 5792 to guard against differences due solely to a change in the sample used. In order to facilitate comparisons across analyses the cut-point of 0/1 specific memories was retained for this analysis rather than re-deriving a new cut-point based on quartiles of the new distribution.
